# The Aegean archipelago: a natural laboratory of evolution, ecology and civilisations

**DOI:** 10.1186/s40709-017-0061-3

**Published:** 2017-02-21

**Authors:** Spyros Sfenthourakis, Kostas A. Triantis

**Affiliations:** 1 Department of Biological Sciences, University of Cyprus, Panepistimiou 1, 2109 Aglantzia, Nicosia, Cyprus; 2 Department of Ecology and Taxonomy, Faculty of Biology, National and Kapodistrian University, Athens, Greece

**Keywords:** Island biogeography, Aegean, Insular communities, Adaptive radiations, Non adaptive radiations, Habitat heterogeneity

## Abstract

The Aegean archipelago, comprising numerous islands and islets with great heterogeneity in topographic, geological, historical and environmental properties, offers an ideal natural laboratory for ecological and evolutionary research, and has been the stage for a very long interaction between human civilizations and local ecosystems. This work presents insights that have been gained from past and current relevant research in the area, highlighting also the importance of the Aegean archipelago as a useful model to address many major questions in biogeography, ecology and evolutionary processes. Among the most interesting findings from such studies concern the role of habitat heterogeneity as the most important determinant of species richness, the development of a new model (Choros) for the species–area–habitats relationship, the mechanistic aspects of the Small Island Effect, the very high rates of species turnover, the lack of a role for interspecific competition in shaping species co-occurrence patterns in most cases, the importance of non adaptive radiation in diversification of several taxa, the insights into the relative roles of vicariance and dispersal in speciation, the understanding of the interplay between human presence and the establishment of exotic species and extinction of indigenous biotas. Concluding, the Aegean archipelago is an ideal stage for research in evolution, ecology and biogeography, and has the potential to become a model study area at a global level, especially for land-bridge, continental islands.

## Background

Almost the entirety of biotic systems on Earth are organized in partially or more strictly isolated entities, i.e. islands, such as marine water bodies surrounded by water of different physicochemical properties, relatively homogeneous habitats surrounded by a broader and different matrix, individual animals for parasites or symbionts, geographic islands, lakes, caves, mountaintops, temporary pools, etc. Therefore, one can perceive the biosphere as consisting of a vast series of variably interconnected islands. This omnipresence of islands in our world, with consequences also in other realms of human endeavors such as art and psychology, is one of the major reasons for the prevalence of island ecology in the development of modern ecological theories. The other is the relative simplicity of biota on geographic islands coupled with their distinct boundaries that facilitate description and analysis of local dynamics.

The Equilibrium Theory of Island Biogeography (ETIB) of MacArthur and Wilson [1, 2] has been the seminal and paradigm-setting work that initiated a vast number of small to large scale research lines aiming to test, refute, correct, add to and/or build on the original theory. A recent review [3] has highlighted major advances in ecological and biogeographical theory made possible by developments in what is generally termed as ‘island biogeography theory’ or more generally ‘island theory’. In addition, the importance of islands for the development of evolutionary theory can be overemphasized, given the role that islands played in Darwin’s and Wallace’s theories. ETIB has been considered as mostly devoid of evolutionary processes, since the main predictors of species richness it encompasses are immigration and extinction rates regarded from an ecological perspective (e.g. extinction refers to loss from an island, i.e. extirpation). Speciation, even though explicitly mentioned by MacArthur and Wilson, has not been part of the mathematical expression of the theory and has been peripherally considered in the first decades after the theory’s publication. More recent developments, mainly the general dynamic model (GDM) of oceanic island biogeography [4], have attempted to also incorporate speciation, via island’s geological age, but we still lack a mathematically coherent general island theory, with speciation incorporated. Nevertheless, the similarities of ETIB and evolutionary models of speciation are long known and, in fact, they have informed each other to a certain extent (see, for example, [5] regarding the effects of immigration history on evolutionary dynamics). In effect, the ETIB can be used to make coarse estimates of speciation probabilities in an insular system, given that high immigration and extinction rates would impede speciation whereas increased isolation coupled with small turnover rates (i.e., large island size) would be expected to promote it. Therefore, the study of island biogeography and ecology is tightly connected to evolutionary processes in space and time.

Within the framework of island theory, islands are broadly classified in three main types, based on their origins and history of formation [6]: oceanic (emerging from sea, never connected with continental masses, most of volcanic origin, e.g. Hawaii, Canary, Galápagos), continental shelf (formed mainly by eustatic changes of sea level, connected with continental masses during glacial periods in the Pleistocene—e.g. Britain, Borneo, New Guinea), and continental fragments (remnants of very old continental masses that were subsequently fragmented, not connected to continental masses since then—e.g. Seychelles, New Zealand, Madagascar). In addition to these three types referring to actual islands, authors often refer to habitat islands which are either fragments of previously larger habitat types (e.g., forest fragments) or uniform habitats surrounded by very different matrices (e.g., mountaintops). Island models are actually applied to all systems consisting of isolated or semi-isolated components, such as lakes, river systems, etc.

Amongst these island types, oceanic islands have gained a central role to the development of theories and their evaluation since the time of Darwin and Wallace. One of the first lessons taught to us by Wallace, decades before MacArthur and Wilson, is that comparisons among different biogeographic regions and archipelagos can provide increased understanding of the processes regulating biodiversity across time and space (see [7]). For the most recent advancements in oceanic island biogeography, see [4, 8–11]. However, oceanic islands are not the majority of true geographic islands. Considering islands with areas larger than 1 km^2^, they represent a 27% of the total number [12]; although an accurate estimation of all the geographical islands of the world is not available, the oceanic ones represent an even smaller fraction of islands globally (using the crude estimation of [13], which is ca. 1 million islands). The vast number of islands on Earth is of the continental shelf type, and any comprehensive theory should also describe patterns and processes of their biota. Despite various efforts, island theory about continental islands is far less mature compared to the oceanic islands theory.

**Fig. 1:**
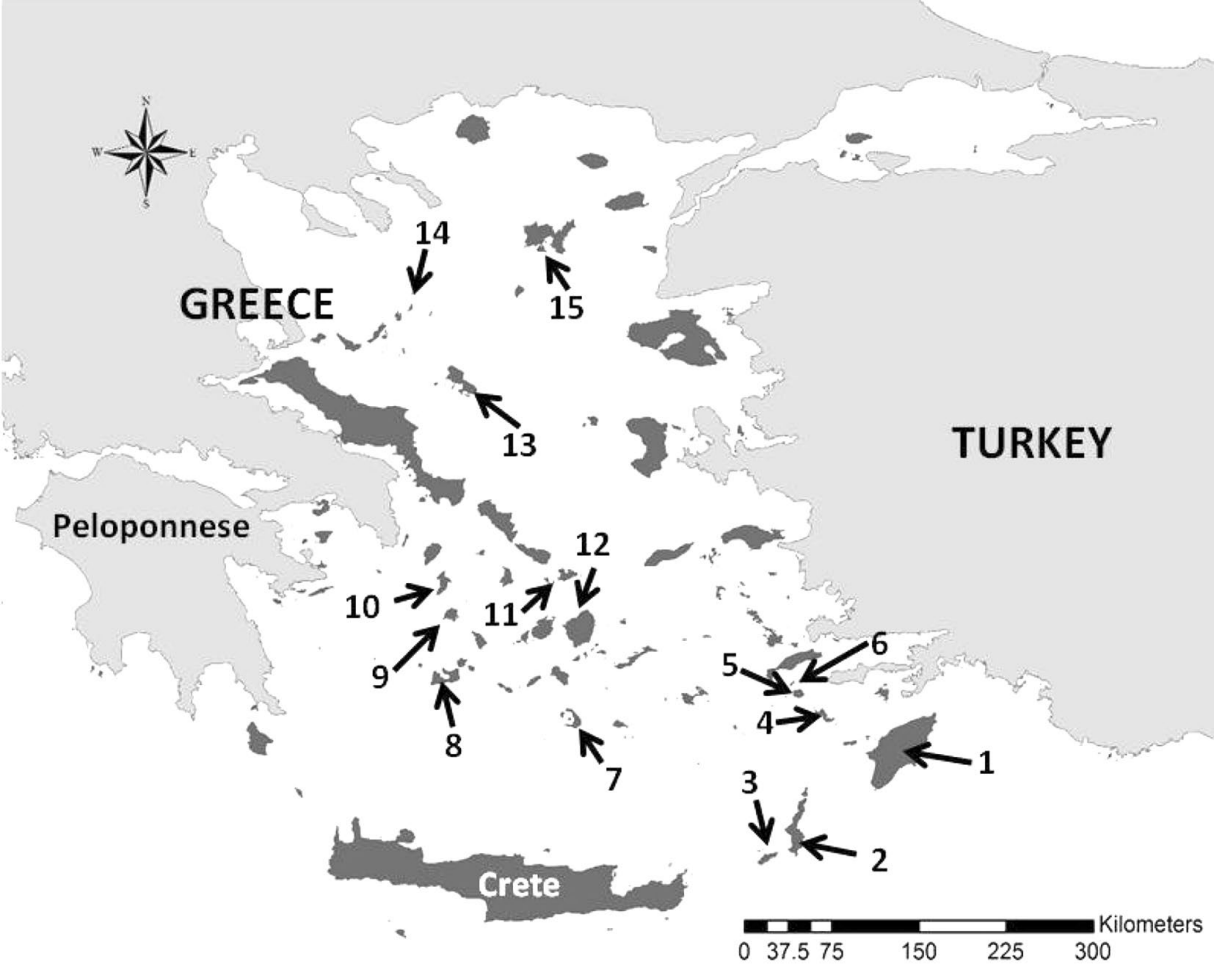
Map of the Aegean archipelago (*darker* islands). *Numbers* indicate islands mentioned in the text. *1* Rhodes, *2* Karpathos, *3* Kasos, *4* Tilos, *5* Nisyros, *6* Gyali, *7* Santorini (Thira), *8* Milos, *9* Serifos, *10* Kythnos, *11* Dilos, *12* Naxos, *13* Skyros, *14* Gioura, *15* Limnos

A typical archipelago of continental islands is the Aegean Sea Archipelago (Fig. 1). Some 7500 islands and islets occur at a variety of isolation levels and topographic features establishing the Aegean Sea amongst the archipelagos with the highest number of islands. The archipelago stands in the center of the conjunction of three continents, namely Europe, Asia, and Africa [14]. Despite the continental origin of the archipelago, quite a small number of islands are of volcanic origin in the Aegean Sea, such as Thira, Nisyros, Gyali etc., as well as a large continental fragment, Crete, which has been isolated for the last c. 6 million years [15]. The Aegean Sea has played an instrumental role to the development of modern island biogeography theory and in fact the development of ETIB itself. In 1964, George E. Watson from Yale University submitted his thesis on the ‘Ecology and evolution of passerine bird on the islands of the Aegean Sea’ [16]. One of Watson’s main conclusions was that in the Aegean islands, habitat diversity is the prime driver of species richness observed. As Rosenzweig [17: p. 217] notes in his seminal book ‘*His work deeply influenced MacArthur, who I know saw a copy before 1965. (He told then how important it was)*’. The Aegean archipelago with other major continental archipelagos of the world such as the Philippines, provide exceptional opportunities for the further development of island theory, especially through the disentangling of under-explored or in need of revisiting theories and patterns. Recently, a list of such theories and patterns has been presented [3]; we herein discuss these under-explored or in need of revisiting ideas under the light of recent developments of the Aegean Sea islands. We also present in short the geological dynamics of the archipelago and the history of humans in the region.

### Palaeogeography of the Aegean

**Fig. 2:**
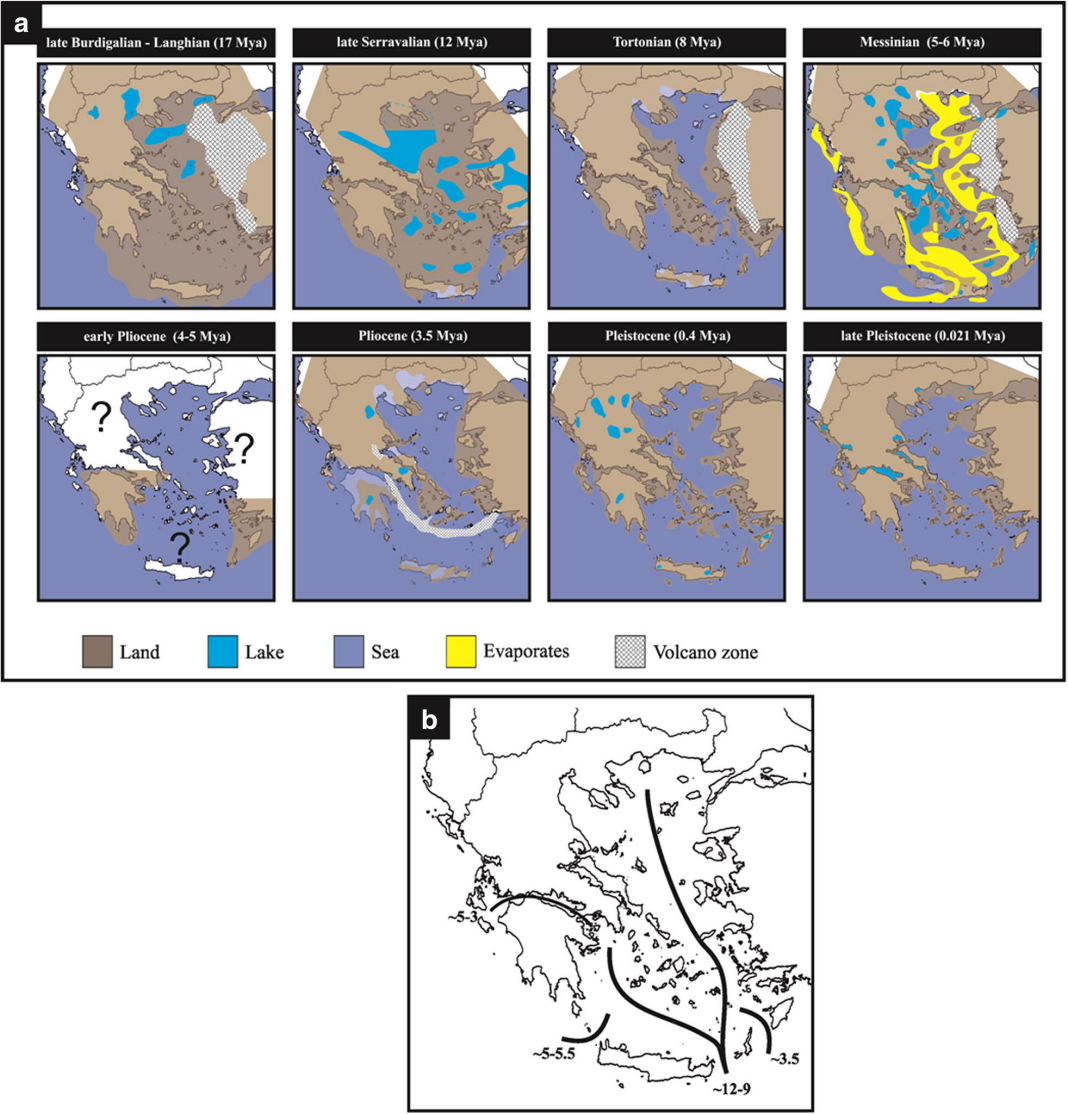
**a** Greece from the Miocene to present, drawn based on the present geography. **b** The main geological barriers in the Aegean. Numbers are in MYA. From [13] (reproduced with permission by John Wiley and Sons)

The geotectonic evolution of the Aegean islands has had a major contribution in shaping the biogeographic patterns of all recent taxa of these areas (see brief review in [15] and references therein). The islands have been formed under the influence of three major forces: tectonism, volcanism, and eustatism. Throughout the Aegean islands history, repeated cycles of connection and isolation from neighboring mainland and insular areas have occurred. These cycles were imposed by the westward movement of the Anatolian plate, the northward movement of the African plate and its subduction under the Eurasian, the Messinian Salinity Crisis, and the sea-level fluctuations during glacial and interglacial periods. Even for those islands that have experienced long-term isolation, fluctuations in size, submergence, and uplift were very common. Crete, for example, has been crushed, folded, pushed, and shattered, producing some of the largest fault-scarp cliffs in Europe. In summary, four main stages in the palaeogeographic evolution of the Aegean can be distinguished. During the first stage (23–12 million years ago, MYA), a continuous land mass (known as Ägäis) was present. In the second stage (12–5 MYA), a slow sea transgression occurred, forming a biogeographic barrier between eastern and central-western parts, known as the mid-Aegean trench (MAT) which most probably remained, albeit much narrowed, even during the Messinian Salinity Crisis (5.96–5.33 MYA), when the Mediterranean Sea almost desiccated. After the refilling of the Mediterranean Sea, and during the third stage (5–2 million MYA), there was extensive fragmentation and a widening of the Aegean Sea. Finally, the fourth stage (during the Pleistocene) involved mainly orogenetic and eustatic sea-level changes. Intensive volcanic activity has also contributed to the formation of several islands, a few of which are purely volcanic (e.g., Nisyros island). On the other hand, the geological evolution of the islands of the Ionian Sea has been quite simple, with most islands becoming isolated from the mainland during the Pleistocene or even more recently (Fig. 2).

### Human presence

**Fig. 3:**
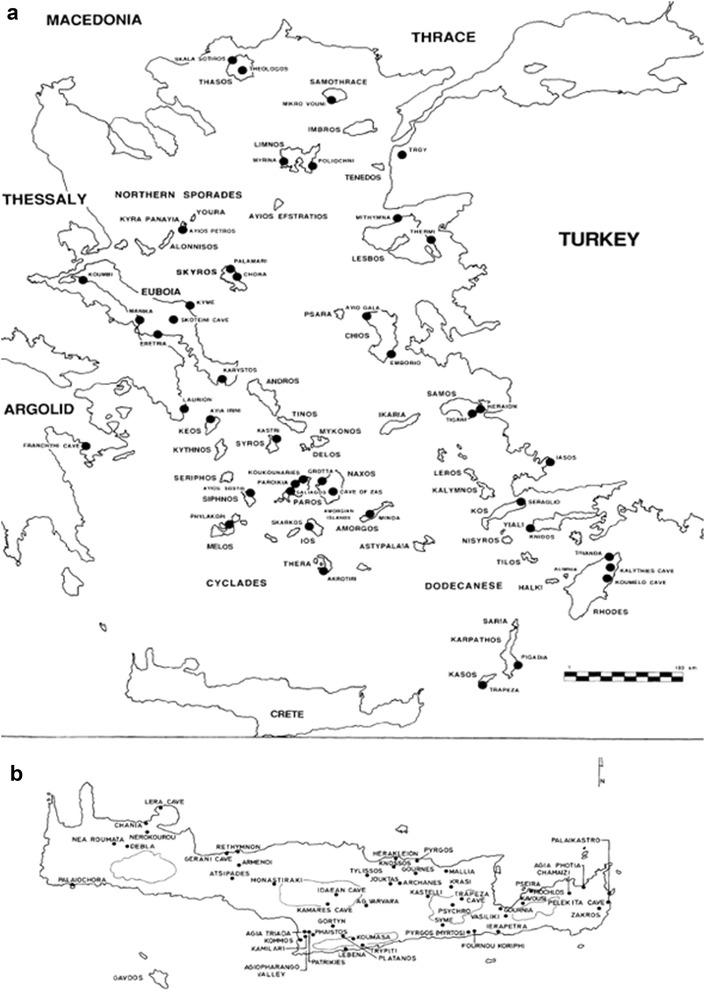
Principal archaeological sites in **a** the Aegean islands except Crete, and **b** Crete. **a** (adapted from [18]), **b** (adapted from [22])

The habitats on Aegean islands have been extensively affected by modern humans, who are continuously present on almost all of them for more than 10,000 years [18]. There is even evidence for Palaeolithic (ca. 130,000 years ago) human settlement, e.g. on Crete [19], but permanent occurrence probably started around the end of the last glacial period. Of course, Aegean islands were not all inhabited all at once [20]. Based on current archaeological evidence the oldest human settlements are present on Limnos (twelfth century BC) and Gioura (eighth century BC) islands at the northern Aegean, and Kythnos island at central Aegean (eighth century BC), whereas humans regularly visited also Milos island to collect obsidian since the eleventh century BC [21, 22]. Dodecanisa islands at the southeastern Aegean, near the Asia Minor coast, were inhabited in Early Neolithic (6500–5800 BC) and most other islands in Late Neolithic (4800–3200 BC) [23, 24] (Fig. 3). The subsequent history of humans in the Aegean was very turbulent, with famous civilizations rising and declining (Minoan, Cycladic, Mycenaean) but also with frequent population movements due to a variety of factors, ranging from piracy to volcanic activity. Whereas larger islands have been used for settlement, cultivation, etc., humans have been exploiting even the smallest islets of the Aegean Sea, mainly for goat grazing but also as temporary residence for fishermen, for religious activities, etc.

In effect, humans have actually (re-)engineered the ecosystems of Aegean islands, changing both their vegetation and vegetation structure (for cultivation, construction, establishing of pasture land, accidental fires, etc.) and their animal communities (by hunting, introduction of domestic animals, vegetation change, etc.). The extent of these changes cannot be fully evaluated, since many species do not leave fossils or other traces. However, it is well established that a few thousand years ago several islands hosted dwarf elephants and/or hippos (e.g., Crete, Rhodes, Tilos, Dilos, Naxos, Kythnos, Serifos, Milos, Kasos) and were covered by dense oak vegetation, while today they are mostly covered by phrygana (dwarf scrubs dominated by low, often cushion-shaped, spiny shrubs) or degraded maquis and the largest mammal present is the badger, *Meles meles*, (with the exception of Samos island where jackals, *Canis aureus*, are still present and until relatively recently one could encounter even leopards, *Panthera pardous saxicolor*, and wild boar, *Sus scrofa*, that swim to the island from Asia Minor due to the very short distance between them).

On the other hand, several traditional human activities do enhance biodiversity on the islands, for example by creating more heterogeneous habitats through small scale cultivations and by using agricultural practices that provide crucial resources to several species (e.g., stonewalls, increased variety of pants, etc.). So, even though we cannot quantify and accurately describe the overall effect of humans on Aegean island ecosystems, anthropogenic factors should always be examined when modern biotic communities are studied. The long and variant human presence in the Aegean Sea, is providing one of the few, if not the only one, system worldwide for the Anthropocene to be studied and understood.

### Modern communities

As we have seen, the palaeogeography and palaeoecology of the Aegean has been very dynamic, with most of modern islands having a long history of repeated merging into larger islands and subsequent fragmentation, of connection and disconnection to neighboring mainland, and of changes in their ecological and topographic features. In consequence, research on the biota of these islands can both help us reveal the geological and evolutionary history of the area and take advantage of established knowledge to provide firm explanations of biodiversity patterns and processes.

Reflecting, more or less, the history of biogeography, such studies have followed two almost parallel lines, with recent attempts towards convergence. One line focuses on insular biotic communities, addressing questions on community composition, similarity, alpha and beta diversity, nestedness, and species co-occurrence patterns, all within the broader context set by the equilibrium theory of island biogeography (ETIB). The second line focuses mostly on evolutionary processes, addressing questions related to speciation, phylogeography and historical biogeography. A larger percentage of studies deal with animal taxa, even though data on plants are much better and abundant and also certain keystone papers have used plants as their study material. In addition, despite the fact that the first seminal paper on marine biogeography has considered depth-related gradients in Aegean taxa [25], the vast majority of published work so far concerns terrestrial taxa. This is mainly driven by the numerous islands of the Aegean Sea, because isolation of terrestrial insular biota, coupled with the large number and variety of Aegean islands, produce a rich pool of patterns and processes that can provide valuable insights into many aspects of biogeography theory.

### Community level

#### Patterns of species richness

What determines the number of species present in a defined region? This is a central question in ecology and a relation between the area of the studied region and the species richness observed therein has been one of the first described patterns in biogeography. Elaborations on the species–area relationships (SARs) have occupied a large bulk of the twentieth century ecological literature, and continue to produce interesting theoretical advancements also in the twenty-first century [26–30]. One can identify two coarse approaches to the explanation of SARs, one using area *per se* as the driving force and the other viewing area as a macroecological descriptor, which captures multiple correlated variables that together determine the available ecological space or ‘environmental heterogeneity’. In the ‘area *per se*’ approaches one can include also explanations relevant to ‘fractal’ structure of communities and/or those addressing specific population distributions, in the sense that area is directly affecting maximum population sizes. The ‘environmental heterogeneity’ approach, implicit in the original equilibrium theory of island biogeography, postulates habitat diversity and habitat heterogeneity (for a clarification of terminology see [31] and Box 1) as the main factor driving species richness, in the sense that larger areas usually host a wider range of habitats, topographic elements etc., enabling thus the occurrence of a wider range of species.

**Box 1:** The ecological Tower of Babel: environmental heterogeneity

The need for a common language among ecologists and closely related disciplines (e.g. biogeography, evolution and conservation biology) has been repeatedly stressed (e.g., [84–89]). This need is becoming ever more urgent. Ecology has matured into a nomothetic science [90] with an ever-expanding literature replete with neologisms and encompassing a growing range of subdisciplines.	
The construction of a common ecological language needs to adhere to two guiding principles: utility and standardization [91]. Unfortunately, many ecological terms lack standardization, directly compromising their utility. Terms have frequently been assigned to different definitions, definitions are used interchangeably (often within the same manuscript), and there is a general lack of precision in the use of terminology. This has led to widespread confusion and conceptual stagnation: an ecological Towel of Babel (the construction of many languages from one).	
The existence of this ecological Tower of Babel has led various authors to plead for a common language (see [88, 92–96]), and a few authors have taken on the challenge (see [94, 95, 97]) and proposed standardized definitions for the most fundamental ecological concepts (environment, community, habitat, biotope, niche). However, even if Looijen’s definitions help resolve the problematic use of concepts like habitat and biotope at a theoretical level, in practice the description and determination of these concepts in real situations is still problematic.	
The practical problem of defining and measuring heterogeneity in nature is especially prominent in fields such as biogeography and macroecology, where much ink has been spent *inter alia* on the role of ‘habitat diversity’ in regulating species richness patterns. In these sub-disciplines a clear, standardized and, most importantly, applicable definition of habitat (and related terms, like biotope, habitat diversity, habitat heterogeneity, ecotope etc.) is of critical importance to advance our understanding of biodiversity regulation. The Aegean islands, with their broad spectrum of natural and human-made habitats provide an excellent system for establishing a coherent terminology in regards to environmental heterogeneity.	

Sfenthourakis [32, 33] found that a measure of habitat diversity was generally a better predictor of island species richness than area or elevation for terrestrial isopods on 42 central Aegean islands. This finding applied both to the total island set and to each of two subsets of island size classes. The important role of habitat diversity (commonly noted by H) has been substantiated by later authors [34, 35] who also proposed a new model (the Choros model) that uses a variable, K, which combines area (A) and habitat diversity (K = A * H). Choros could be perceived as the space ‘experienced’ by species assemblages of a taxon. This model proved to be a better, or at least equally good, predictor than area or habitat diversity alone in the majority of cases where it was tested [34]. Nevertheless, the measurement of habitat diversity remains a tricky issue in ecology, and no consensus among researchers has been reached on both theoretical and practical issues related to this concept (see Box 1). In a contribution to this discussion by the authors [36, 37], furthering the approach developed in [32, 33], habitat diversity has been viewed as taxon-based with its estimation demanding information on the ecological requirements of the taxon studied. The authors also showed an important role of the relative contribution of generalist and specialist species in insular assemblages in shaping species richness patterns, especially those related with what has been known as Small Island Effect (SIE: when species richness does not respond to area in very small islands—see [6]). Keystone habitat types seem to play a crucial role in the existence of a SIE in a specific system. This work triggered an exchange of opposing views on both the SIE and habitat diversity, which led to a better clarification of the relevant concepts.

Fattorini [38] analyzed a data set of Aegean tenebrionid beetles and found that island area accounted for most variability in species richness compared to distances from continental areas; he identified a clear faunal discontinuity between western plus central Aegean and eastern islands, which is explained as evidence of the importance of Pleistocene island configurations in determining present distributions, while at the same time he concluded that tenebrionids of Aegean islands are relictual. At the same time he assumed that most tenebrionid species colonized Aegean islands by means of land-bridges during Pleistocene falls of sea level. These largely incongruent conclusions were probably the result of using an incomplete data set, since tenebrionid beetles have not been consistently recorded on many Aegean islands. This problem also undermines his results on spatial patterns, latitudinal diversity gradients, biodiversity hotspots, community nestedness and co-species occurrence analyses of the same data set [39–42], despite the fact that some of his conclusions are in line with those of the analysis of other, better studied, taxa (see relevant discussion in [43]).

One crucial component of the equilibrium theory of island biogeography, species turnover, has received less attention over the years, mainly due to the difficulty in documenting actual turnover rates that may need long time periods of recordings. Nevertheless, there have been some estimations of turnover based on empirical data in insular systems that are studied for several decades (e.g. [44, 45]). One such opportunity has been offered by studies of plants on small islands in the northwestern Aegean, where comparable data were collected in the 1970s and the 1990s. Given the very small size of the islands, and thus also of the corresponding plant communities, the ca. 20-year interval between recordings was considered adequate for an insightful estimation of turnover rates.

Panitsa et al. [46] documented one of the largest values of turnover rate ever found (mean relative turnover rate per islet = 2.06 species per islet per year). Even if one takes into consideration phenomena like pseudoturnover and cryptoturnover, the values are indeed impressive and show that insular communities, especially of small islands and islets, change continuously in accordance with the main premises of the equilibrium theory of island biogeography. It is important to note that even many of the species widely present in both study periods (regarded as ‘core’) were found on different islets in the second study period. What is more impressive in this study, and something that can be considered as even stronger evidence in favor of the equilibrium theory, is that community-level properties of the islets remained almost identical despite the large change of species composition. In particular, the slopes of species–area and species–elevation regressions and islet by islet floral similarity indices (Jaccard’s) were almost identical in 1974 and 1994, while nestedness and co-occurrence levels were also very similar in the beginning and the end of the 20-year period.

#### Community assembly

Insular communities have also played an important role in the development of community-level theories that relate to what has been generally addressed as ‘assembly rules’, such as those related with community nestedness and species co-occurrences, as well as patterns of alpha- and beta-diversity.

Nestedness related research on Aegean islands has contributed to this discussion, mainly through the analysis of a few taxa for which detailed information on both local and ‘regional’ communities has been made available, such as terrestrial isopods, land snails, centipedes, birds and plants [47–50]. Most island groups in the Aegean Sea exhibit high to medium levels of nestedness, as expected by their continental character. Patterns of nestedness in most groups are affected to some degree by the palaeogeographic history of the archipelago. This history has led to a compartmentalization of various islands in groups whose biota retains its signal. The historical effects, though, have been mixed with those of habitat diversity, producing a more complicated modern pattern [47]. In highly mobile taxa, such as birds, though, the historical signal is not strong and nestedness patterns are mostly regulated by area effects [49].

One line followed by researchers on species co-occurrences has focused on detecting significant patterns of ‘segregations’ (species that tend to occur together in the same site less often than expected by chance) and/or ‘aggregations’ (species that tend to occur together more often than expected by chance) in species per sites (islands, communities, samples, etc.) matrices. This line of research has also been extended to the study of interaction networks, such as hosts–parasites and pollinators–plants networks. Similarly to nestedness analysis, the study of co-occurrences relies heavily on the application of ‘null models’, since standard statistics usually cannot provide significance estimations. The main reason for this is that there is no a priori expected distribution of occurrences in a matrix.

The basic assumption behind this approach is that if interspecific competition plays an important role in community assembly, then its effects should be traceable in the distribution patterns of interacting species, so that competing species should tend to avoid each other more than expected by chance. Expectedly, there has been some controversy over methods, algorithms and metrics, and the results of analyses are often contradictory. Contributions from Aegean islands to this discussion, albeit limited in number, have lead to some interesting findings. Sfenthourakis et al. [51] were able to test patterns of species co-occurrence at different scales, both among sampling stations on each island and among different islands, and came to the conclusion that coexistence is more common than mutual exclusion, but in any case, the few apparently deviating species associations are probably due to factors such as the habitat structure and historical events, and not of direct biotic interactions (e.g. competitive exclusion). In another contribution, the same authors applied a method that could test for possible causal mechanisms on co-occurrence patterns and found that evidence in favor of competition is very limited in Aegean terrestrial isopods [52]. The lack of strong evidence for interspecific competition was corroborated also by extensive analysis of data sets from other regions, taxa and taxonomic levels (i.e., focusing on congeneric species). Similar results were found by Gotelli and Ulrich [53] and Pitta et al. [54]. The latter work revealed that the famous ‘assembly rules’ proposed by Diamond [55], which lead to very productive research, were actually based on the very few exceptional matrices where evidence for competition is indeed present! The same pitfall led Sanderson et al. [56] to support competition as an important determinant of matrix-based patterns, as they based their work on these same exceptional matrices.

### The evolutionary perspective

Evolutionary diversification on island systems has been central in the development of evolutionary theory, since the multiple isolation of an initially common genetic pool, i.e., of the first immigrants, can lead to quick allopatric speciation. Adaptive radiation on archipelagos has been shown in several taxa and is a fairly well understood process, mostly when taking place in island groups. Besides adaptive radiation, though, some authors have also proposed another process that may lead to quick diversification of insular populations, which is assumed as not been triggered by selection, and is referred to as non-adaptive radiation. Even though the documentation of non-adaptive radiation is difficult, since one can always assume a role of selective forces that researchers failed to identify, there are several cases where this process seems to be very plausible. Among the first such cases that were quite robustly documented came from taxa distributed among Aegean islands. Several decades ago, the Swedish botanist Hans Runemark proposed such a process, which he called ‘reproductive drift’, for the differentiation of the *Nigella arvensis*-group in the Aegean. This process was actually a case of genetic drift that led to non-adaptive divergence of the plant’s populations on different Aegean islands. More recent research by the team of Hans Peter Comes (Salzburg University) has provided additional genetic evidence in favor of non-adaptive radiation in this group. Comes et al. [57], after detailed molecular studies on the *N. arvensis* complex, stressed the importance of allopatry and genetic drift in speciation at various temporal and spatial scales. The recent (Late Pleistocene) radiation of this group is strongly conditioned by palaeogeographic factors, but shifts in breeding system (selfing) and associated isolating mechanisms have also played an important role. On the other hand, founder effects were not found to be significant, probably because the continental character of the Aegean islands has enabled high levels of dispersal where processes like niche pre-emption due to a long established resident flora are more important. Bittkau and Comes [58] found no significant effect of Quaternary climatic oscillations on accelerated speciation rates of *N. arvensis* complex, that are probably due to increased opportunities for allopatric speciation within the palaeogeographically complex Aegean archipelago and the Late Pleistocene changes in sea level.

Another strong case for this process had been made also by Gittenberger [59] who proposed that the patterns of divergence among several *Albinaria* populations in Greece support a non-adaptive radiation scenario. On the other hand, Giokas et al. [60] argued in favor of selection-driven morphological variation of *Albinaria*. The relationship between morphological variation, clade divergence, adaptive and non-adaptive radiation in this taxon still remains ambiguous.

An additional study that favored non-adaptive radiation, after detailed and combined morphometric and phylogenetic analyses is the one by Parmakelis et al. [61] on the evolutionary differentiation of the land snail genus *Mastus* in the Aegean. Furthermore, Kamilari and Sfenthourakis [62] showed that non-adaptive radiation has been responsible for the differentiation of certain characters in the very variable isopod species *Armadillo tuberculatus* that is represented by a different morph on almost each island of the central and southern Aegean. At the same time, a closely related taxon, *A. officinalis* that is distributed in the same region, remains almost invariable in both morphological and molecular characters (SS: personal data).

The relative role of vicariance and dispersal in speciation patterns has been addressed by several authors. A study on intraspecific variation of the damselfly *Platycnemis pennipes* in the Aegean region [63] concluded that its current distribution patterns have been formed by an interplay of dispersal and vicariance, also affected by the species’ ecological features. This study downplayed the role of vicariance as the main driver of diversification, as presumed by many authors at that time. Hausdorf and Hennig [64] identified geography at different geological periods, from current to Miocene, as main determinants of butterfly, snail, isopod, reptile and tenebrionid beetle communities.

Nevertheless, these studies were mainly based on morphological and/or community composition data, whereas molecular evidence has renewed interest in major vicariance events as major drivers of evolutionary differentiation in several taxa (see review in [15]). In addition to phylogeographic studies that usually explore patterns at a large scale, some authors have focused on small scale phenomena, such as [65] who explored gene flow among small islands at a local group in order to evaluate the role of vicariance in the divergence of lizards. Nevertheless, we cannot resort to single explanations, since different taxa may be affected by different factors and even the same taxon maybe affected by different factors in different periods of its evolutionary history [15].

The variety of examples in the Aegean can provide a promising source of information for further studies in the reasons behind differential evolutionary potential and rates among different taxa, in the role of ecological specialization in evolution, in variability of dispersal abilities among closely related taxa, etc.

### Island ecology

Studies of animal or plant communities and assemblages on islands may have provided significant insights into the various aspects of island biogeography theory, but what about the ecology of insular systems? What have we learned on the way species and communities interact within each island and with the abiotic components of the environment? This line of research has not been followed by many researchers. After an early ambitious effort in the ‘80 s of a team lead by the late Prof. Matsakis, aiming to a detailed study of various ecological aspects of typical Mediterranean-type ecosystems on the island of Naxos, only a few other attempts towards this goal have been made. The ‘Naxos group’ consisted of a good part of the young generation of enthusiastic Greek ecologists that soon afterwards formed the core of the current ecological community in the country. The scientific output of this pioneering project, though, remained relatively poor [66 and references therein]. More recent work on various ecological subjects, such as plant–pollinator interactions [67–69] or the relationships among diversity, productivity and stability/resilience of ecosystems, such as in the framework of the BIODEPTH experiment [70–72], may have been conducted on islands but are not addressing specific aspects of insular ecology as such. As a consequence, questions regarding ecological processes that may vary between island and mainland habitats, such as the role of coastal habitats and halophilic communities as filters, the role of sea-spray and local climatic variability due to topographic factors for insular communities, interspecific interactions in the absence of large predators and/or within simplified food webs, intraspecific interactions in the face of restricted resources, etc., remain largely under-explored in the Aegean Sea. Of course, there are a few exceptions, such as the exploration of plant competition after perturbation [73], the evaluation of environmental factors affecting plant communities on insular mountainous habitats [74], or the role of marine subsidies in modulating the ‘island syndrome’, as expressed through variation in the reproductive biology among local populations on islets [75].

In the line of research more pertinent to succession studies and to the building of novel communities on new islands, Dimopoulos et al. [76] showed that the formation of local pioneer vegetation on the small volcanic islands of the Thira group is controlled to a significant extent by factors such as the distribution of ashes after volcanic eruptions and the physicochemical properties (e.g. nutrient content) of soil formed by the deposition of ashes, as well as by the substrate age as communities ‘mature’ through saturation of their local species pools. The occurrence of very recent volcanic islands within the Aegean, with known history of formation, provides invaluable opportunities for case studies on colonization processes, pioneer species, community assembly, etc. Similarly, the frequent perturbations by humans offer case studies in the role and processes of species invasion in local communities. For example, Dimitrakopoulos et al. [77] studied plant species invasions in burnt and unburnt Mediterranean grasslands and found an important role of species richness and composition in regulating invasibility at local scales, regardless of effects from disturbance by fire. Also, Vila et al. [78] conducted field experiments on different Mediterranean islands, including Aegean ones, and found that island ecosystems are generally resistant to invaders (but remarkably not to *Oxalis pes*-*caprae*) and differences in invasibility among islands might depend more on propagule pressure or factors acting at late life-history stages. In general, invasions in insular Mediterranean ecosystems seemed to be idiosyncratic and to depend strongly on water availability.

The complex processes that have structured Aegean archipelago and the recent formation of many islands and islets offer the stage for a particularly active interplay between ecological and evolutionary processes. Studies on the evolutionary ecology of Aegean organisms have been conducted mostly in reptiles, revealing gigantism in an insular endemic lizard (*Podarcis gaigeae*) and its relation with intraspecific competition [79] and dietary niche divergence [80]. Relaxed predation on lizards has been also shown to be a factor in phenotypic divergence among islands [81], but genetic drift has been also invoked [82] to explain coloration variability among insular populations in combination with local selection. Marshall et al. [83] investigated camouflage patterns of the Aegean wall lizard (*Podarcis erhardii*) against predatory birds in five island populations and found that these have been adapted to the local substrate of each island, regardless of the age of island separation, showing that anti-predator adaptation to different environments may contribute to evolutionary divergence within species, thus to ecological speciation.

## Conclusions

Overall, studies on biota of the Aegean islands have yielded useful insights into several crucial questions in biogeography, ecology and evolutionary biology. Here is a brief summary of the most important such findings:

- Habitat heterogeneity is the most important determinant of species richness, with a key role of major habitats;
- A new model (Choros) for the species–area–habitats relationship has been developed;
- Very high species turnover rates have been shown at a temporal scale of a few decades, but community level properties remain stable within the same time period;
- Community nestedness is mostly affected by palaeogeographic/historic factors, especially for taxa with low mobility; in contrast, communities of mobile taxa, especially birds, are mainly affected by current geographic and climatic factors;
- Interspecific competition does not play any role in shaping species co-occurrence patterns in the vast majority of cases;
- Non adaptive radiation is an important process in driving diversification even in very short time scales, manifested in parallel with deterministic processes;
- The relative roles of vicariance and dispersal in evolution should be evaluated at a case-by-case basis, with no general rule being possible.

Nevertheless, up to now, ecological and evolutionary studies in the Aegean Sea are still limited and scarce and have been restricted to a relatively small number of taxa. We lack information on many insect and other species-rich invertebrate groups (e.g., Nematoda), but even the potential of the better known taxa has not been exploited in full.

The Aegean archipelago is an ideal stage for research in evolution, ecology and biogeography, and has the potential to become a model study area at a global level, especially for land-bridge, continental islands. Of critical importance is the understanding of the interplay between human presence, establishment of exotic species and extinction of indigenous biotas. The Aegean Sea, being one of the most touristic destinations globally, is continuously affected by human activities thus the monitoring of the changes caused can inform and guide global policies. The topographic, palaeogeographic, climatic and ecological properties of the archipelago, combined with the very long presence of humans and the excessive number of islands, provide a unique combination for biogeographers and evolutionary ecologists to develop and test theories and models of biodiversity’s establishment and regulation.
